# Emerging Innovations in the Treatment of Fuchs Endothelial Corneal Dystrophy: A Narrative Review

**DOI:** 10.3390/medsci13040238

**Published:** 2025-10-22

**Authors:** Magdalena Niestrata, James Jackson, Shehnaz Bazeer, Mingya Alexa Gong, Zahra Ashena

**Affiliations:** 1 Department of Ophthalmology, Institute of Ophthalmology, University College London (UCL), London WC1E 6BT, UK; alexa.gong.19@ucl.ac.uk; 2 Data Governance and Statutory Returns, University of East London (UEL), London E16 2RD, UK; james@j3j.co.uk; 3 Moorfields Eye Hospital NHS Foundation Trust, London EC1V 2PD, UK; 4 Barking, Havering and Redbridge University Hospitals NHS Trust, Romford, London RM1 2BA, UK; zashena@nhs.net

**Keywords:** Fuchs endothelial corneal dystrophy, DMEK, DWEK, endothelial cell therapy, synthetic cornea, ROCK inhibitor, artificial intelligence, review

## Abstract

Fuchs endothelial corneal dystrophy (FECD) is the leading cause of endothelial failure requiring keratoplasty in industrialised nations. Descemet membrane endothelial keratoplasty (DMEK) has become the gold-standard surgical therapy, yet it is constrained by limited donor tissue and a steep learning curve. This narrative review summarises current and emerging therapeutic strategies for FECD. We describe conventional endothelial keratoplasty and its outcomes, tissue-sparing procedures such as descemetorhexis without endothelial keratoplasty (DWEK) and quarter-DMEK, regenerative approaches including cultured endothelial cell injection and synthetic corneal substitutes, and adjunctive innovations ranging from Rho-associated kinase inhibitors to artificial intelligence-assisted diagnostics. Challenges surrounding donor shortages, variable clinical outcomes, regulatory hurdles and cost are critically appraised. We conclude by outlining future directions that are likely to combine advanced surgical techniques with cell-based and biomaterial solutions to deliver accessible, long-term restoration of vision for patients with FECD.

## 1. Introduction and Methodology

### 1.1. Introduction

Fuchs endothelial corneal dystrophy (FECD) is a degenerative eye disorder characterised by the progressive loss of endothelial cells in the cornea, which are crucial for maintaining corneal clarity by regulating fluid balance. As these cells deteriorate, fluid accumulates within the cornea, resulting in corneal oedema, clouding, and subsequent vision loss. This condition, although genetic, often manifests later in life making it more common in older adults.

Contemporary global meta-analysis estimates that approximately 7.33% of adults over 30 years of age are affected by Fuchs’ endothelial corneal dystrophy, with prevalence projected to rise from around 300 million in 2020 to 415 million by 2050 [1].

Recent research has shed more light on FECD’s complex pathophysiology. Notably, the common late-onset form of FECD is associated with a non-coding trinucleotide repeat expansion in the TCF4 gene, leading to toxic RNA aggregates and oxidative stress in endothelial cells [2]. This results in the hallmark formation of guttae (focal collagen excrescences on Descemet’s membrane) and accelerates endothelial cell loss. Early-onset familial FECD, in contrast, often stems from missense mutations (e.g., in COL8A2), causing more aggressive endothelial failure [2]. Ultimately, as endothelial pump function declines, corneal hydration increases and oedema worsens, triggering the visual decline observed in FECD. Understanding these disease mechanisms underscores the development of effective treatment modalities.

With the global population ageing, the incidence of FECD is expected to rise, leading to an increase in cases of corneal decompensation, particularly after common procedures like cataract surgery. This decompensation occurs when the already compromised endothelial cells are unable to recover from the stress of surgery, exacerbating the condition and accelerating the need for intervention.

Currently, the gold standard treatment for FECD is Descemet membrane endothelial keratoplasty (DMEK). This advanced surgical procedure involves replacing the diseased endothelial layer with healthy donor tissue. DMEK is preferred for its ability to restore vision and corneal clarity more effectively than older techniques, such as penetrating keratoplasty (PK) and Descemet stripping automated endothelial keratoplasty (DSAEK), due to its more precise anatomical restoration and lower rates of graft rejection.

However, a major challenge in the widespread adoption of DMEK is the limited availability of donor corneal tissue. This shortage is particularly acute in regions like the United Kingdom, where the demand for donor tissue often exceeds supply. The scarcity of donor corneas not only restricts the availability of DMEK but also delays treatment for many patients, exacerbating the progression of FECD. This situation has prompted significant research into alternative treatments and innovative approaches to managing FECD, aiming to reduce reliance on donor tissue and improve patient outcomes [3].

### 1.2. Methodology

**Figure 1:**
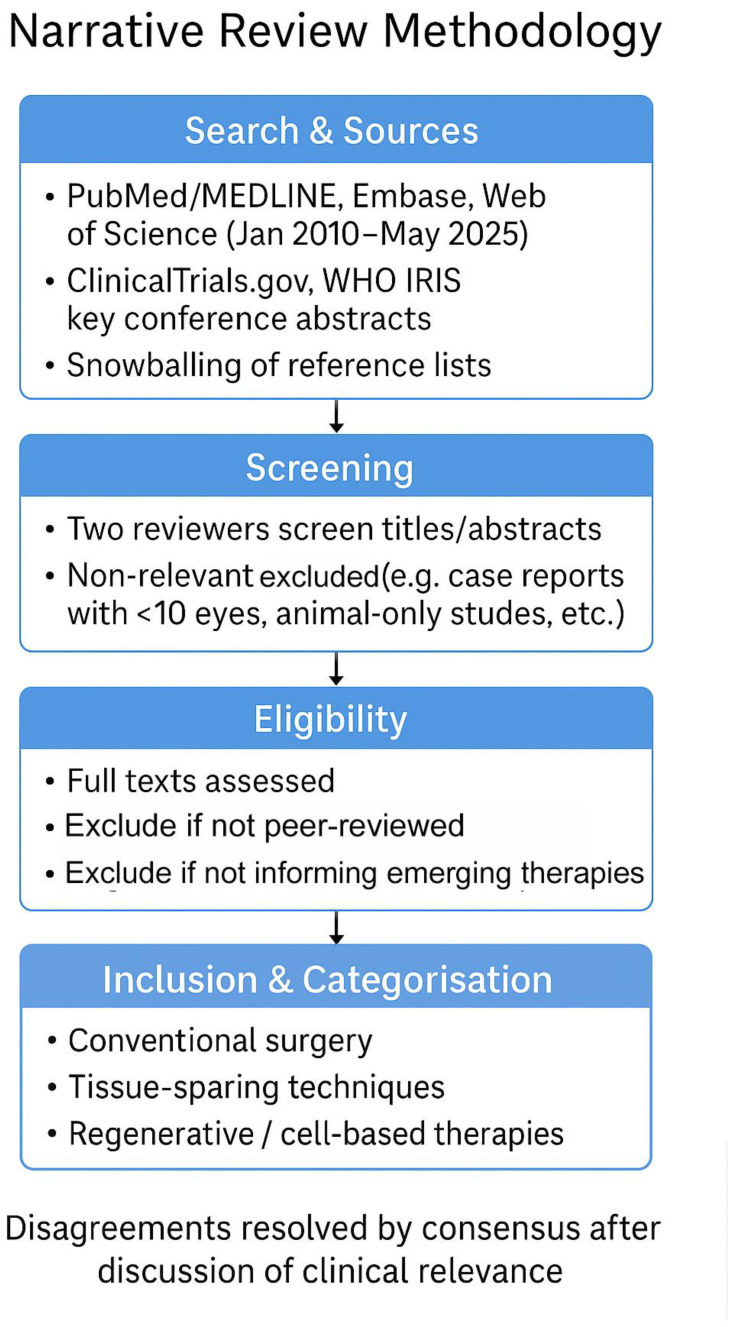
Diagram of the methodology.

This was a narrative review. We searched PubMed/MEDLINE, Embase and Web of Science for articles published between January 2010 and May 2025 using combinations of the terms ‘Fuchs endothelial corneal dystrophy’/‘FECD’ with ‘DMEK’, ‘DSAEK’, ‘DWEK/DSO’, ‘quarter-DMEK’, ‘Rho-kinase inhibitor’, ‘endothelial cell injection’, ‘iPSC’, ‘keratoprosthesis’, and ‘EndoArt’. ClinicalTrials.gov and WHO IRIS were screened for ongoing or policy-related work, and key conference abstracts were considered when they reported first-in-human or practice-changing data. Two authors independently screened titles/abstracts and full texts; disagreements were resolved by consensus after discussion of clinical relevance. We prioritised peer-reviewed original studies (randomised or prospective series ≥10 eyes), systematic reviews and pivotal translational studies directly informing current or emerging therapies; single case reports and animal-only studies were excluded unless they introduced a novel device or approach. Reference lists of included papers were hand-searched to capture additional studies (snowballing), and the scope was iteratively refined to ensure balanced coverage of four domains: conventional surgery, tissue-sparing techniques, regenerative/cell-based therapies and adjunctive pharmacologic/technological innovations. Study quality was appraised using a modified SANRA checklist for narrative reviews, shown as per Figure 1.

## 2. Conventional Surgical Management

### 2.1. Introduction: Prevention of Corneal Decompensation After Cataract Surgery

In patients with known Fuchs’ dystrophy, steps can be undertaken during cataract surgery to protect the endothelium and reduce the risk of its decompensation post-operatively. These include using dispersive viscoelastic to coat the endothelium prior to phacoemulsification (soft-shell technique), employing femotosecond-assisted cataract surgery when available to lower the ultrasound energy required, and using techniques that minimise the amount of ultrasound energy used, such as direct chopping techniques [4,5] However, taking all those precautions only reduces and does not eliminate the list of endothelial decompensation post-operatively.

### 2.2. Penetrating Keratoplasty (PK)

Penetrating keratoplasty, the historical benchmark for endothelial disease, replaces the full-thickness cornea with a donor button. It reliably restores optical clarity but often yields suboptimal vision due to high irregular astigmatism, demands lengthy suture management, and carries elevated long-term rejection and wound-dehiscence risks. Consequently, visual rehabilitation is protracted and unpredictable, making PK largely a last-line option for advanced or multi-layer pathology [6].

### 2.3. Descemet Stripping Automated Endothelial Keratoplasty (DSAEK)

**Figure 2:**
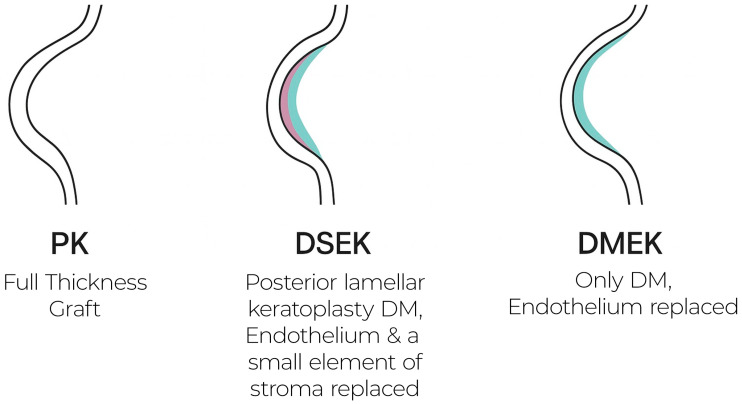
Schematic of keratoplasty techniques.

DSAEK supplants only the posterior stroma, Descemet membrane, and endothelium. Its 80–150 µm lamellar graft markedly reduces rejection relative to PK and shortens recovery, as shown in Figure 2. Nonetheless, the residual stromal interface can produce haze and higher-order aberrations, limiting best-corrected visual acuity to ~20/30–20/40 for many patients [7]. Interface fluid, graft folds, and thickness-related hyperopic shift remain recognised drawbacks.

### 2.4. Descemet Membrane Endothelial Keratoplasty (DMEK)

DMEK transplants solely the donor Descemet membrane and endothelial monolayer, achieving near-anatomic restoration of the posterior cornea. Key advantages include

- Superior visual acuity and quality—the ultrathin (≈10 µm) graft induces negligible astigmatism and eliminates stromal interface haze, yielding faster recovery, fewer higher-order aberrations, and better contrast sensitivity than PK or DSAEK [8]
- Lower complication profile—rejection rates are the lowest among keratoplasty techniques, and endothelial cell loss parallels or improves upon DSAEK at 1 year [8]

Limitations and challenges

- Technical demands—successful trephination, unfolding, and centration of the fragile graft require advanced surgical skill; learning curves typically span 25–50 cases [9].
- Graft detachment and rebubbling—the delicate membrane is prone to early post-operative separation, necessitating intracameral air/gas reinjection (“rebubbling”) in up to 20% of eyes, prolonging recovery [10], although this rate declines with surgeon experience [9].
- Donor-cornea scarcity—global tissue shortages, acute in low- and middle-income regions and still felt in high-income nations such as the UK, restrict access and extend waiting times; logistics of preservation and transport further complicate supply [3].

DMEK currently defines the gold standard for endothelial keratoplasty, delivering the best visual outcomes with the lowest rejection risk. Widespread adoption, however, hinges on expanding surgeon training and alleviating donor-tissue shortages, spurring interest in tissue-sparing and regenerative alternatives. Eye banks in many regions continue to operate below pre-pandemic recovery levels, with interruptions to the donor pipeline caused by staffing deficits, reduced elective surgery scheduling, and ongoing limitations in tissue retrieval and screening infrastructure. Furthermore, national variations in death-to-preservation time, cold-chain dependence, and quality-control regulations lead to significant disparities in tissue utilisation and wastage rates. This fragile supply chain has direct implications for clinical outcomes: prolonged wait times often allow FECD progression to severe decompensation, reducing the success of subsequent keratoplasty and narrowing the therapeutic window for tissue-sparing interventions. [11]

Recent policy guidance from the World Health Organisation (2024) [12] recommends decentralising advanced therapy manufacturing and eye-bank services through “Regional Advanced Therapy Laboratories” to improve resilience, reduce intercontinental dependence, and ensure equitable access to vision-restoring procedures globally. These strategies are particularly critical as the global demand for endothelial keratoplasty is projected to exceed the donor supply by more than fivefold in the coming decade.
**Feature****PK****DSAEK****DMEK****Typical Visual Outcome**Ultimately can restore clear cornea, but visual rehabilitation is often prolonged and variable. Post-operative astigmatism patients’ vision is variable, dependant on many factors including surgical technique. Often, additional treatments are required to optimise vision further, including glasses, contact lenses, refractive laser, and lens replacement [6,7,13]Improved vision with less astigmatism than PK. Best-corrected acuity is typically around 20/30–20/40 Snellen in many cases, limited by interface haze. Rarely achieves 20/20 Snellen due to graft thickness [6,9,10]Excellent visual outcomes, approaching normal corneas. A majority of eyes reach ~20/25 Snellen or better clarity when fully healed, and many attain 20/20 Snellen. Eliminating the stromal interface gives DMEK superior contrast and acuity [8,9,14]**Complications and Risks**Highest rejection risk of all techniques (historically ~15–20% within a few years). In a recent 10-year study, PK had ~13% rejection by year 10, higher than DMEK. Requires sutures—risk of wound dehiscence, suture infections, and significant post-op astigmatism. Graft failure rate increases over time as endothelium decompensates [6,7]Moderate rejection risk (~5–10% in first 1–2 years; ~19% by 10 years). Some risk of graft dislocation, though lower than DMEK (thicker graft adheres more firmly). Main drawbacks are interface-related: residual stromal layer can cause optical aberrations and mild haze. Overall complication profile is improved over PK, but not as low as DMEK [6,9]Lowest rejection rate (~1% or less in first 2 years; ~10% by 10 years—still lowest of the three). No full-thickness wound -> no wound dehiscence issues. Key complication is graft detachment: ~10–20% of cases require a repeat air bubble (“rebubbling”) to reattach the delicate graft in early post-op. Surgery has a steep learning curve, which can initially increase complication rates (e.g., higher detachment if surgeon is inexperienced) [6,8,10,15]**Donor Tissue Requirement**One full-thickness donor cornea per transplant (1 donor:1 patient). No tissue sparing—a scarce resource is used entirely for one surgery [13]One partial-thickness donor graft per surgery (still essentially 1 donor:1 patient). Does not reduce overall donor demand (though leftover anterior cornea might be used for partial-thickness anterior grafts in other contexts) [9,13].One thin endothelial layer graft per surgery (1 donor:1 patient in standard practice). Does not save donor tissue unless using novel “split graft” techniques. Note: Emerging variants like quarter-DMEK divide one donor Descemet’s membrane into 2–4 pieces, treating multiple patients with one donor, but this is not yet widespread standard practice [9,16,17]**Typical Recovery Time**Longest. Visual recovery is slow—often 12 to 18 months before sutures are removed and vision stabilises. A large study found the median time to reach ~20/40 vision after PK was ~37.9 months (about 3 years), reflecting the prolonged rehabilitation from both wound healing and refractive corrections [6,7].Intermediate. Faster than PK, but still requires months. Median ~12 months to reach ~20/40 vision in one 10-year study. Many patients have functional vision by ~3–6 months post-op, but improvement can continue up to a year as the interface settles and any residual refractive error is addressed [6,9].Shortest. Rapid recovery of clarity—often ~4–8 weeks to achieve significant vision improvement. Median time to ~20/40 vision is ~7.8 months, but unlike PK/DSAEK, a substantial fraction of DMEK patients see well (20/25 or better) as early as 2–3 months post-op [8,9,10].

## 3. Tissue-Sparing Strategies

### 3.1. Descemetorhexis Without Endothelial Keratoplasty (DWEK/DSO)

Descemetorhexis without endothelial keratoplasty (DWEK), or Descemet-stripping only (DSO), entails excising a 4–6 mm zone of guttae-laden Descemet membrane from the visual axis while leaving the underlying posterior stroma bare. By forgoing a donor graft, the procedure depends entirely on the patient’s own peripheral endothelial cells to migrate centripetally, repopulate the defect and restore corneal deturgescence. The approach is therefore best suited to eyes with early-to-moderate FECD in which the peripheral cell density remains above roughly 1000 cells mm^2^ and guttae are confined centrally [18,19]. 

Recent in vivo confocal microscopy data from a prospective interventional case series (n = 34 eyes) provide new insights into endothelial dynamics post-DSO with and without additional treatment with ROCK inhibitors, agents that promote endothelial cell migration. In this study, 76% of eyes received adjunctive topical ROCK inhibitor, ripasudil, while 24% did not. Remarkably, 94% of ripasudil-treated eyes achieved corneal clearance, compared to only 75% in the non-ripasudil group, with mean clearance achieved within six weeks in the treated cohort. Confocal images revealed enhanced endothelial cell migration at the leading edge and reformation of a cohesive mosaic pattern—morphological changes not observed in untreated eyes [20]. These findings underscore how ROCK inhibitor therapy accelerates healing, supporting hypothesis-driven patient selection and tailored postsurgical management.

Complementing these physiological insights, the ongoing DETECT II randomised controlled trial (NCT05275972) is comparing DSO with adjunctive ripasudil against DMEK in early-to-moderate FECD. With planned enrolment of 60 participants across seven sites in the USA, the trial aims to establish whether DSO-R matches DMEK in visual outcomes and endothelial function at 12 months [21]. Its results will be pivotal in determining whether DSO with ROCK inhibition can become a standard, tissue-sparing alternative in FECD management. Clinical results are encouraging: published case series report corneal clearance in 60–85% of eyes, with responders achieving a final best-corrected acuity near 20/25 once oedema resolves. Regeneration, however, is slow: objective clarity often takes three to six months to re-establish, a timeline patients must accept in advance. Non-responders—most commonly those with underestimated peripheral endothelial compromise or impaired migratory capacity—can still undergo endothelial keratoplasty later without penalty. The principal unresolved questions concern durability beyond five years and the risk of late central decompensation as the peripheral reservoir ages. Notwithstanding these uncertainties, DWEK offers a genuinely tissue-sparing, rejection-free option that completely sidesteps global donor-cornea shortages, provided that careful patient selection is observed [14,22].

### 3.2. Quarter-DMEK and Other Mini-Grafts

Mini-graft techniques—most notably quarter-DMEK—aim to maximise scarce donor tissue by dividing a single Descemet membrane–endothelium roll into two to four fragments, each transplanted into a different recipient. After a standard descemetorhexis, the surgeon introduces one quarter of the membrane and positions it eccentrically so that the endothelial lattice overlies the host’s denuded zone. Early prospective reports describe corneal clearance in more than 90% of treated eyes, with six-month visual acuities indistinguishable from full-size DMEK; endothelial cell loss and rebubbling rates are likewise comparable when meticulous centration and unfolding are achieved [16]. 

The chief advantage of quarter-DMEK is obvious: it multiplies donor yield at a stroke, an attractive proposition for programmes hamstrung by tissue scarcity. Optical quality is preserved because the graft remains ultrathin and interface-free. The downsides lie in surgical complexity, with surgical skill again playing a role in outcomes [23]. Handling, inserting and orienting a smaller, more delicate scroll is technically demanding, and off-axis placement can introduce subtle higher-order aberrations if centration relative to the pupil is imperfect. In addition, the long-term survival and cell-loss kinetics of divided tissue remain to be mapped out in larger cohorts with follow-up extending beyond five years. Nevertheless, early data suggest that mini-grafts could become a pragmatic bridge between current donor limitations and future cell-based or synthetic solutions [24], provided that ongoing refinements in instrumentation and technique continue apace, although large-scale data of outcomes beyond 5 years are not yet available [25].

DWEK offers a compelling, truly tissue-sparing option for carefully selected FECD eyes, obviating donor dependence and graft-related complications. Broader adoption, however, will depend on reliable predictive markers of endothelial migratory capacity and further long-term outcome data to confirm sustained corneal clarity and the criteria for the need for simultaneous ROCK inhibitor treatment. Likewise, mini-graft techniques offer a bridge to future solutions by maximising donor utility, though surgical refinements and prospective outcome studies are ongoing.

## 4. Regenerative and Cell-Based Therapies

A growing body of translational research now focuses on restoring corneal endothelial function without relying on donor grafts. Three complementary strategies are gaining momentum: direct cell injection, scaffold-assisted cell delivery and fully synthetic keratoprosthetic substitutes.

### 4.1. Cultured Endothelial Cell Injection Therapy

Kinoshita and colleagues pioneered a protocol in which donor corneal endothelial cells (CECs) are enzymatically dissociated, expanded ex vivo with a Rho-associated kinase (ROCK) inhibitor and then injected into the anterior chamber [26]. In their multicentre phase-I/II trial, 11 of 15 treated eyes maintained a clear cornea three years post-operatively—an overall success rate of 73%—with mean endothelial cell densities stabilising above 1000 cells mm^2^ and central corneal thickness normalising within two months. Subsequent Japanese cohort studies have reproduced 70–80% of treated FECD eyes at 3 years, consistent with these results. Recently, a 5-year follow-up found normal corneal function maintained in 10 of 11 eyes (91%) that received cultured cell injections, confirming durability [27,28]. 

The approach offers several advantages: (i) one donor cornea can seed vials sufficient for scores of injections, dramatically amplifying tissue yield [26]; (ii) the procedure is suture-less and minimally invasive; and (iii) immunological rejection is rare. Remaining hurdles include optimising cell-culture protocols to prevent fibroblastic transformation, proving lot-to-lot consistency for regulatory approval and confirming durability beyond five years [29]. The body of clinical evidence and patient numbers are currently low; however, the results obtained are encouraging, and it is worth watching this space in the years to come.

### 4.2. Scaffold-Supported Endothelial Constructs

Where cell injection alone may be insufficient—particularly in eyes with irregular posterior stroma—researchers are evaluating biomaterial scaffolds that deliver CECs as an engineered monolayer. Hydrogel membranes composed of collagen type-I, gelatin methacrylate or decellularised Descemet membrane support tight-junction formation and Na^+^/K^+^-ATPase expression in vitro, then peel away or biodegrade in vivo after engraftment. Preliminary rabbit and porcine studies show rapid corneal deturgescence and uniform cell coverage, and a first-in-human pilot using a fibrin–gelatin carrier reported stromal clearing in four of five FECD eyes at 12 months [30]. Key questions now concern mechanical strength during handling, optical transparency, immunogenicity of xenogenic components and scaling manufacture under current good-manufacturing-practice (cGMP) conditions.

### 4.3. Synthetic (Keratoprosthetic) Substitutes

When human grafts have failed or are not available, fully synthetic keratoprostheses may offer an off-the-shelf alternative. The EndoArt^®^ device—an ultrathin, transparent hexafluorosilicone membrane “artificial endothelial layer” developed by EyeYon Medical—is composed of a 50 µm-thick, 6–6.5 mm diameter posterior corneal implant, which mimics endothelial fluid-barrier function and adheres directly to Descemet’s membrane via a peripheral suction ring, thereby reducing corneal oedema by physical compression of the cornea. A first-in-human European series showed prompt oedema resolution in three of four eyes, though two required repositioning due to early dislocation [31]. It offers a promising alternative to traditional corneal endothelial transplantation for patients with chronic corneal oedema not responding to other treatments and high-risk eyes. Across multiple small studies, EndoArt^®^ consistently reduced central corneal thickness (average drop ~150–300 µm) and improved best-corrected visual acuity by about 0.3–1.2 logMAR within 3 months. Rebubbling was frequently required (~48–80% of cases) due to detachment risk [31]. A 4.5-year follow-up case series showed sustained implant adherence in 9 of 10 eyes, stable corneal thickness reduction, visual gains in 60% of eyes, and no device-related adverse events [32]. In addition, the device has shown promising results in patients with advanced glaucoma and previous glaucoma surgeries, which pose difficulty for traditional keratoplasty. These findings highlight EndoArt’s potential as a durable, biocompatible endothelial prosthesis, especially for patients with previous graft failures, ocular comorbidities, lowering the chance of success of human keratoplasty, or in areas lacking donor tissue access [31,32].

Outstanding concerns include long-term endothelial-side biofouling, risk of stromal melt, interface opacification and the need for secondary anchoring surgery should the prosthesis dislodge [3]; however, the early results are promising in ongoing studies, notably that preliminary clinical experience with intrastromal placement of EndoArt^®^ has demonstrated reliable adhesion and sustained corneal clarity, hinting that refinements in implantation technique could address several of these limitations [33]. Recombinant human collagen, cross-linked with carbodiimide or polyethylene glycol, has likewise been fashioned into acellular “corneal buttons” with promising optical clarity but limited long-term human data [34].

### 4.4. iPSC-Derived Endothelial Cell Therapy

In addition to using primary donor cells, researchers are exploring induced pluripotent stem cells (iPSCs) as a source of corneal endothelium. In 2024, a first-in-human trial transplanted iPSC-derived endothelial cell grafts into patients with bullous keratopathy (corneal oedema post-surgery in the absence of Fuchs’ dystrophy). After one year, the treated corneas had significantly improved clarity and visual acuity with no immune rejections or serious adverse events [35]. This approach could be beneficial for Fuchs’ dystrophy patients as well and potentially provide an unlimited supply of transplantable endothelial cells, eliminating donor dependency. However, it raises new safety considerations—for instance, comprehensive genomic analyses of the iPSC-derived cells revealed a de novo mutation in one cell line (in the EP300 gene), albeit with no observed clinical effect at 1 year [35]. Long-term follow-up is needed to ensure iPSC therapies pose no tumorigenic or late failure risks, as current studies are only in early stages after non-human trials [35]. Nonetheless, the success of this trial demonstrates the feasibility of replacing donor corneas with laboratory-grown cell substitutes, heralding a promising frontier in regenerative treatment of FECD.

Regenerative therapies for FECD are transitioning from bench to bedside. Cell-based approaches—whether injecting cultivated donor endothelial cells or transplanting scaffold-supported cell sheets—have demonstrated the ability to re-establish corneal clarity in the majority of early trials [27]. These strategies dramatically amplify the utility of donor tissue and even hint at a future of donor-independent treatment through iPSC-derived cells [28]. Early results are encouraging, but challenges like long-term cell survival, phenotypic stability, and regulatory approval remain. If these hurdles are overcome, such regenerative therapies could fundamentally reduce reliance on donor corneas in FECD.

## 5. Adjunctive Pharmacologic and Technological Innovations

### 5.1. Rho-Associated Kinase (ROCK) Inhibitors

ROCK signalling governs cytoskeletal contractility in CECs. Topical ripasudil and netarsudil, as well as intracameral fasudil, have been shown to stimulate cell migration and proliferation in animal models. Clinically, adjunctive twice-daily ripasudil after DWEK halved the median clearing time—~12 weeks down to ~4 weeks—in a prospective Japanese cohort, with no significant intraocular-pressure spikes or endothelial toxicity [13,36]. ROCK inhibition is also integral to the culture medium used for cell injection therapy, mitigating anoikis and enhancing seeding efficiency by up to five-fold [17]. Ocular hyperaemia and mild, transient verticillate keratopathy constitute the most frequent side-effects [25].

### 5.2. Advanced Imaging and Artificial Intelligence

High-definition anterior-segment optical coherence tomography (AS-OCT) and confocal specular microscopy deliver micron-scale maps of guttae density, corneal thickness and endothelial cell morphometry. When coupled to convolutional neural-network classifiers, these datasets can grade FECD severity with ≥95% accuracy, anticipate need for keratoplasty within two years and even predict post-DMEK rebubbling risk [15,37]. Deep-learning-enabled pachymetry heat-maps further permit automated monitoring of corneal deturgescence after emerging therapies, shortening follow-up visits and standardising outcome metrics across centres [38,39]. The principal implementation barriers are capital cost, data-protection compliance and the creation of ethnically diverse training sets to avoid algorithmic bias.

Adjunctive innovations are enhancing FECD treatment outcomes and decision-making. Pharmacologically, Rho-kinase inhibitors have shown they can accelerate corneal endothelial healing—for example, eyes treated with topical ripasudil after DSO clear oedema in a fraction of the time of observation alone [25]. Such adjuvants may expand the pool of patients eligible for non-graft therapy. Meanwhile, advances in imaging and artificial intelligence are improving diagnostics and post-operative care: high-resolution OCT combined with AI can accurately grade FECD severity and even predict which patients will soon require keratoplasty. These technologies offer more personalised and proactive management of FECD, as well as the possibility to risk-stratify patients, which is important in the context of tissue shortages and waiting times, complementing the surgical and regenerative treatments discussed above [40].

## 6. Challenges, Knowledge Gaps and Future Directions

The logistical fragility of the donor-tissue supply chain remains the most immediate barrier to equitable keratoplasty worldwide. Even before the COVID-19 pandemic, fewer than one cornea was available for every seventy patients who needed one; pandemic-related travel restrictions and eye-bank shutdowns exacerbated the gap, and global eye-bank output in 2024 has yet to return to its 2019 peak [11]. Although cell injection and scaffold constructs multiply tissue yield, their manufacture currently depends on sophisticated clean-room facilities that are concentrated in high-income regions. Technology-transfer blueprints, such as the WHO-endorsed “Regional Advanced Therapy Laboratories” model published in 2024, are therefore essential to prevent a widening treatment divide [12,41].

Economic evaluations are another blind spot. A recent Canadian Monte Carlo cost-utility analysis projected the lifetime costs and quality-adjusted life years (QALYs) of injectable human corneal endothelial cell (hCEC) therapy compared with DSAEK and DMEK in Ontario, applying a willingness-to-pay threshold of $50,000 per QALY gained. The model suggested that hCEC therapy could be cost-effective relative to current keratoplasty techniques when accounting for procedural wait times, lifetime effectiveness, and discounted costs [42]. However, no peer-reviewed study has yet incorporated the capital costs of cGMP facility construction or the price of regulatory compliance, which now includes whole-genome sequencing of each master cell bank in both Europe and the United States.

Long-term safety data for regenerative products are likewise scant. Late-onset endothelial pump failure has been reported in two Japanese eyes five years after injection therapy, prompting speculation about gradual phenotypic drift [28]. For synthetic keratoprostheses, biofilm-related stromal melt remains the chief late complication; a French registry analysis identified a 9% explant rate by year four for the EndoArt^®^ device, predominantly due to chronic interface inflammation [43].

New biological technologies introduce additional uncertainties. Ex vivo CRISPR-Cas9 base editing has successfully corrected the COL8A2 Q455K mutation in donor CECs without detectable off-target edits [44], but regulatory agencies are still debating whether edited cells should be classified as “advanced therapy medicinal products” or “genetically modified organisms,” a distinction that carries major implications for trial design and post-marketing surveillance. Three-dimensional bioprinting, now capable of generating posterior corneal discs with embedded micro-fluidic channels that mimic Descemet stromal undulations [45], faces the dual hurdles of reproducibility and optical clarity when scaled beyond small animal models.

Digital innovation will shape future service delivery. Large language models integrated into electronic medical records can now pre-populate referral letters and flag progressive corneal oedema on serial AS-OCT scans with 97% specificity [39]. Yet data governance frameworks remain inconsistent; only 43 of 194 WHO member states had fully implemented health-data-protection statutes by the end of [46]. Establishing federated-learning consortia—where encrypted, locally stored imaging datasets train shared algorithms—offers one route to harness AI while respecting regional privacy laws.

Finally, climate resilience is emerging as a non-trivial consideration. Most endothelial-cell culture media still require continuous cold-chain transport; severe heatwaves in 2023 led to a ten-day suspension of corneal shipments into southern Europe, underscoring the importance of thermostable lyophilised formulations now entering phase-I testing [46].

In summary, the next decade will have to reconcile scientific possibility with manufacturing scalability, regulatory clarity, fiscal sustainability and climate-robust logistics. Without deliberate planning, the risk is that transformative therapies will remain geographically siloed, mirroring the inequities that have long plagued conventional corneal transplantation.

## 7. Conclusions

Fuchs endothelial corneal dystrophy is evolving from a disorder treated predominantly by cornea transplantation to one managed through a graduated arsenal of tissue-sparing, regenerative and technology-enabled interventions. DMEK retains unmatched optical performance but will not, by itself, meet global demand. The field is therefore pivoting towards donor-independent options: DWEK for carefully selected early disease, quarter-DMEK to stretch donor supply, cultured-cell (and stem cell-derived) injections and scaffolded constructs to industrialise endothelial replacement, donor-independent options (including potential iPSC-derived tissues) and keratoprosthetic membranes for eyes unsuitable for biological repair. Parallel developments in ROCK-modulating pharmacology and AI-facilitated imaging promise to shorten recovery, personalise therapy and harmonise outcome measurement.

Yet the road from proof-of-concept to population-level impact is long. Achieving durable corneal clarity without unforeseen toxicities will require multicentre, randomised non-inferiority trials with at least five-year follow-up, alongside health-economic studies that capture real-world manufacturing and distribution costs. Regulatory agencies must craft pathways that encourage innovation while ensuring genomic, immunologic and cybersecurity oversight. Above all, stakeholders must embed equity into the development pipeline—through technology-transfer partnerships, tiered-pricing models and adaptation of protocols to resource-limited settings; lest the benefits of these breakthroughs accrue only to patients in privileged regions.

If these scientific, logistical and ethical challenges can be met, the prospect of eliminating preventable blindness from endothelial disease becomes plausible rather than aspirational. Such a paradigm shift will not result from any single discovery but from sustained, multidisciplinary collaboration among surgeons, cell biologists, materials scientists, data scientists, policy-makers and patient advocates. With that collective endeavour, the transition from scarce grafts to scalable, personalised corneal rehabilitation may well define the next era of ophthalmic care.

## Data Availability

No new data was created or analyzed in this study.
